# Application of Titanium Mesh in the Early Treatment of Flail Chest

**DOI:** 10.1155/2024/8213995

**Published:** 2024-11-13

**Authors:** Gaofeng Liu, Li Zhou, Chen Li, Junying Cao, Yong Zhang, Sujuan Cui, Yan Liu, Yanbin Xu, Yongjie Zang, Meiming Yang, Qingyuan Li

**Affiliations:** ^1^Department of Cardiothoracic Surgery, The 988th Hospital of PLA Joint Logistics Support, Zhengzhou 450042, Henan Province, China; ^2^Xinxiang Medical University, Xinxiang 453003, Henan Province, China

**Keywords:** bone-healing, flail chest, hemodynamics, pulmonary function, titanium mesh

## Abstract

**Objective:** To investigate the effect of the titanium mesh on flail chest and bone healing from clinical and animal experiments.

**Methods:** Clinical experiment: 24 patients with flail chests in our hospital from January 2020 to January 2023 were prospectively selected and divided into control and titanium mesh groups according to different treatment plans and basic data-matching principles, with 12 cases in each group. The control group was treated with conservative external fixation, and the titanium mesh group was treated with titanium mesh fixation. The clinical efficacy index, visual analog scale and blood gas indexes and hemodynamic indexes of the two groups of patients were recorded. Chest CT and pulmonary function and life quality were examined after operation. Animal experiment: The flail chest sheep were treated conservatively with a titanium mesh, and the expression of bone-healing-related proteins was detected.

**Results:** The mechanical ventilation time, drain indwelling time, ICU observation time, and hospital time in the titanium mesh group were significantly shorter than those in the control group (*p* < 0.05). The PaO_2_, CVP, FVC, FEV1, MVV, and life quality of the titanium mesh group were significantly better than those of the control group after operation, and the visual analog scale, PaCO_2_, CI, ELWI, and the proportions of atelectasis, thoracocyllosis, and consolidation tardive after operation were significantly lower than those of the control group (*p* < 0.05). The expressions of BMP2, IGF-1, VEGF, and PDGFD in the rib tissue of titanium mesh sheep were higher than those of control sheep at 4 weeks after operation (*p* < 0.05).

**Conclusion:** Titanium mesh is a safe and effective treatment for flail chest, which can improve pain, blood gas, hemodynamic indexes, and pulmonary function and promote fracture healing.

## 1. Introduction

Flail chest is a severe chest trauma characterized by multiple rib fractures, thorax softening, and abnormal breathing, which seriously affects respiratory function [1, 2]. Effective fixation of rib fracture, maintenance of fracture stability, and restoration of thoracic integrity are keys to the treatment of flail chest and the promotion of recovery of patients [3, 4]. With the continuous development of internal fixation materials, internal fixation has been adopted to repair rib fractures in clinical practice, which can shorten the treatment cycle and improve the quality of life of patients [5]. At present, the sandwich method of double polymer mesh composite bone cement is commonly used in the world for bone chest wall reconstruction [6], but it still has some shortcomings such as limited movement of the chest wall after reconstruction, unable to be applied in the case of infection, and affecting postoperative radiotherapy or X-ray examination. In addition, the use of self-tissue materials for the reconstruction of bone thorax has the disadvantages of complicated operation, great trauma, and limited materials, which is not conducive to the early rapid reconstruction of bone thorax. Therefore, it is of great significance in the treatment of flail chest to find a fixed material that can effectively prevent the softening of the chest wall and abnormal respiratory movement, without adverse reaction and rejection, and is easy to set.

Titanium mesh is a kind of biological material, which has the characteristics of stable physical and chemical properties, good histocompatibility and depression molding fixation, etc., and has been applied in fracture treatment [7, 8]. Studies have shown that the surface of the titanium mesh is smooth and has many small gaps, which is conducive to blood circulation between the two sides of the bone and promotes bone regeneration. It has been reported that the application of the titanium mesh can increase the success rate of bone regeneration in bone defects by 50%. However, it has not been studied in the treatment of flail chest.

Therefore, this study compared the clinical effects of external fixation and titanium mesh fixation in the treatment of flail chest from the aspects of recovery, pain, hemodynamics, lung function, complications, and mortality and observed the effect of titanium mesh on bone healing through animal experiments.

## 2. Materials and Methods

### 2.1. Patient Samples

**Figure 1:**
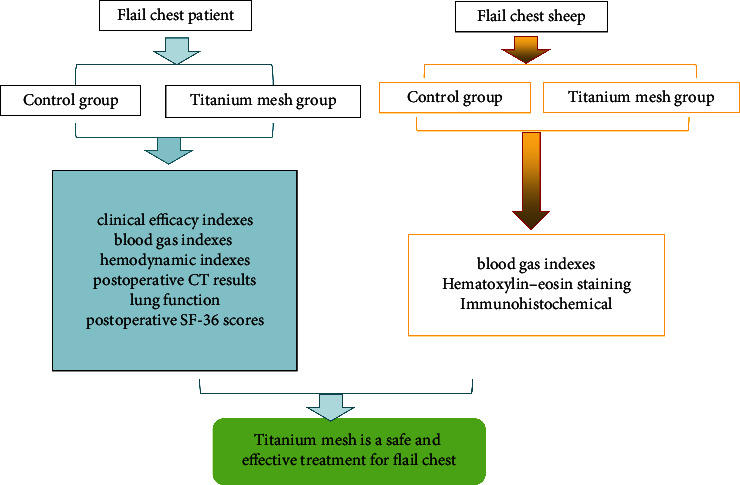
Experimental flow diagram.

**Figure 2:**
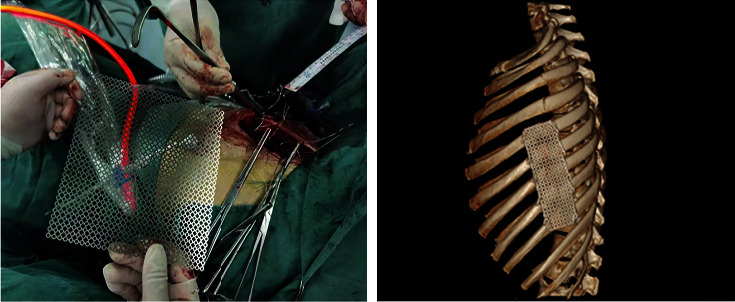
Titanium mesh fixed flail chest.

Twenty-four patients with flail chests admitted to our hospital from January 2020 to January 2023 were prospectively selected. The inclusion criteria were as follows: (1) Multiple rib fractures combined with abnormal breathing were clinically diagnosed as flail chest; (2) no surgical contraindications; (3) they were treated by the same team of doctors and returned to the doctor regularly after surgery; and (4) patients and their family members gave informed consent to the treatment plan, and the compliance was good. Exclusion criteria were as follows: (1) single rib fracture, (2) accompanied by serious heart, lung, brain, and other important diseases of the organs, (3) accompanied by neurological and mental disorders, and (4) medical records and follow-up data are incomplete. The study design is depicted in Figure 1. The patients were divided into control and titanium mesh groups according to different treatment plans and basic data-matching principles, with 12 cases in each group. The control group was treated with conservative external fixation: According to the location of the patient's rib fracture, the guard plate was fixed to the chest wall at the rib fracture. The titanium mesh group was fixed with titanium mesh (Figure 2): The patient underwent a small incision thoracoplasty, and the two ends of the rib fracture were fixed with the titanium mesh to stabilize the bone thorax. This study has been approved by the Committee on Ethics of Medicine, The 988th Hospital of Joint Logistics Support Forces, PLA (Approval Number: 988YY2020060LLSP).

### 2.2. Clinical Data Collection

1. Clinical case observation was performed every day after surgery, and mechanical ventilation time, drain indwelling time, intensive care unit (ICU) observation time, and hospital stay of patients in the two groups were recorded.
2. Pain assessment was performed before operation, 24 and 48 h after operation, and blood gas indexes and hemodynamic indexes were determined. The visual analog scale (VAS) score was used to evaluate the pain of the patient. The full score was 10 points. The higher the score, the more severe the pain of the patient. A blood gas analyzer was used to detect the partial pressure of oxygen (PaO_2_) and partial pressure of carbon dioxide (PaCO_2_). Blood oxygen saturation (SaO_2_) was detected by the blood oxygen monitor. Hemodynamic parameters included cardiac index (CI), central venous pressure (CVP), and extravascular lung water index (EVLWI).
3. Chest CT and lung function were examined one or 2 months after operation. Chest CT: atelectasis, thoracicyllosis, and consolidation tardive were independently judged by three senior thoracic surgeons based on physical examination and chest CT findings, and the diagnosis was recorded as consistent with that of more than two physicians. Forced vital capacity (FVC), forced expiratory volume in the first second (FEV1), and maximal voluntary ventilation (MVV) were measured by a pulmonary function apparatus.
4. The life quality of the patients was evaluated by SF-36 score 2 months after operation. The SF-36 score has 36 items representing eight dimensions, namely physical function (PF), role physical (RP), bodily pain (BP), general health (GH), vitality (VT), social function (SF), role function (RE), and mental health (MH), and the total score of each dimension is 100. The score directly reflects the health status.

### 2.3. Animal Model Establishment and Intervention

**Figure 3:**
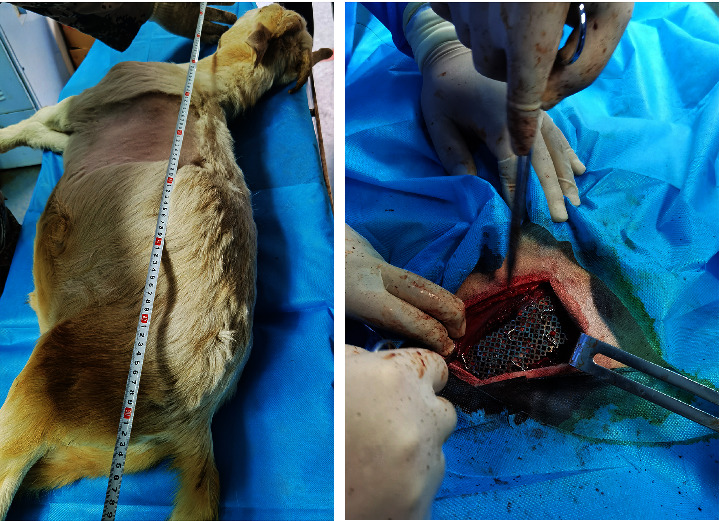
Titanium mesh fixed flail chest sheep.

Twelve healthy adult sheep, Animal License Number: SYXK (Henan) 2016-0001, were used. After 1 week of adaptive feeding, the sheep were free to feed and drink water during the experiment. General anesthesia was administered by the intramuscular injection of sumianxin (0.1 mL/kg, Changsha Beit Biotechnology Research Institute Co., Ltd.). Sheep were fixed on the operating table on their side, skinned, disinfected, and covered with towels, assisted by the breathing machine, and operated under sterile conditions. The muscle layer was cut to the outer side of the rib through the posterolateral incision, and the arteries, veins, and nerves at both ends of the ribs of 5, 6, and 7 were ligations respectively. Cut the ribs of 5, 6, and 7 successively to form three free ribs about 3 cm long to form the flail chest. After the titanium mesh group was molded, the titanium mesh was selected, appropriately cut, and fixed with steel wire to the rib fracture end of the flail chest, and rapid bone reconstruction thorax surgery was performed (Figure 3). Routine anti-inflammation and anti-infection treatment were given every day after operation. The limb movement, eating, and wound healing of the animals were observed, and the walking condition of the animals after the removal of external fixation was observed. One week after surgery, the suture was removed 2 weeks after operation. In the control group, the self-made thoracic drainage tube was reserved in the chest after sufficient hemostasis, muscle skin was sutured layer by layer, and the chest band was bound and fixed with pressure.

### 2.4. Hematoxylin–Eosin (HE) Staining

The blood gas indexes were measured before operation, 24 and 48 h after operation. Then, 4 or 8 weeks after operation, the sheep were euthanized with an overdose of sodium pentobarbital (80–90 mg/kg, Sinopharm Group Chemical Reagent Co., Ltd.), and specimens of the ribs of 5, 6, and 7 were obtained. The specimens were fixed with 10% neutral formaldehyde buffer for 24 h, the 5% nitric acid solution was decalcified for two or 3 days, and then, diluted ammonia water was added for 1 h, and the water was rinsed with running water for 24 h until the pin was easily penetrated. Dehydrated with concentration gradient ethanol, xylene transparent, paraffin-embedded, and decalcified tissue sections were prepared on a pathological micrograph, dewaxed xylene-impregnated slices, and washed with water. Then, HE staining was used and observed by the optical microscope.

### 2.5. Immunohistochemical (IHC) Analysis

After dewaxing the tissue sections, gradient ethanol was rehydrated, and the primary antibodies of bone morphogenetic protein 2 (BMP2), insulin-like growth factor 1 (IGF-1), vascular endothelial growth factor (VEGF), and platelet-derived growth factor D (PDGFD) were added proportionally, and the negative control sections were treated with PBS, reacted at room temperature for 30 min, rinsed with PBS, incubated at 37°C for 30 min, and rinsed with PBS. DAB droplets were added to the slices and incubated for 3 to 15 min. The sections were fully rinsed, restained, dehydrated, transparent, sealed, and observed under an optical microscope. The staining intensity and the proportion of positive cells were determined. Staining intensity score (0–3 points): 0 points for nonstaining, one point for yellow, two points for brown-yellow and three points for yellow-brown. Percentage of positive cells score—< 10%, 10%–40%, 40%–70%, > 70%, were rated 0–3. The final result was the sum of the staining intensity score and positive cell proportion score.

### 2.6. Statistical Analysis

SPSS 19.0 statistical software was used for data analysis. Statistical data were expressed ($\overset{¯}{x}\pm s$), the independent sample *t* test was used for comparison between two groups, statistical data were expressed as percentages, and the *χ*^2^ test was used for comparison between groups. *p* < 0.05 indicates a significant difference.

## 3. Results

### 3.1. Comparison of Clinical Efficacy Indexes Between the Two Groups

There were no significant differences in age, gender, BMI cause of injury, injured position, associated injury, and number of injured ribs between the two groups (Table 1, *p* > 0.05). The mechanical ventilation time, drain indwelling time, ICU observation time, and hospital stays in the titanium mesh group were significantly shorter than those in the control group (Table 2, *p* < 0.05). VAS scores in the titanium mesh group were significantly lower than those in the control group at 24 and 48 h after operation (Table 2, *p* < 0.05).

### 3.2. Comparison of Blood Gas Indexes and Hemodynamic Indexes Between the Two Groups

There was no significant difference in the preoperative blood gas index and hemodynamic index between the two groups (Table 3, *p* > 0.05). PaO_2_ and CVP in the titanium mesh group at 24 and 48 h after operation were significantly higher than those in the control group, and PaCO_2_, CI, and ELWI were significantly lower than those in the control group (Table 3, *p* < 0.05). There was no significant difference in SaO_2_ levels between the two groups at 24 and 48 h after operation (Table 3, *p* > 0.05).

### 3.3. Comparison of Postoperative CT Examination and Lung Function Between the Two Groups

The proportions of atelectasis, thoracicyllosis, and consolidation tardive in the titanium mesh group at 1 and 2 months after operation were significantly lower than those in the control group (Table 4, *p* < 0.05). FVC, FEV1, and MVV indexes of the titanium mesh group were significantly better than those of the control group at 1 and 2 months after operation (Table 4, *p* < 0.05).

### 3.4. Comparison of Postoperative Quality of Life Between the Two Groups

Except for RE, the SF-36 scores of the titanium mesh group were statistically higher than those of the control group (Table 5, *p* < 0.05).

### 3.5. Comparison of Blood Gas Indexes Between the Two Groups of Sheep

To verify the effect of titanium mesh on the flail chest, we used titanium mesh the on flail chest sheep injury model (Figure 1). There was no significant difference in PaO_2_, PaCO_2_, and SaO_2_ levels between the two groups before and after operation (Table 6, *p* < 0.05).

### 3.6. Histopathological Examination of Ribs of Two Groups of Sheep

**Figure 4:**
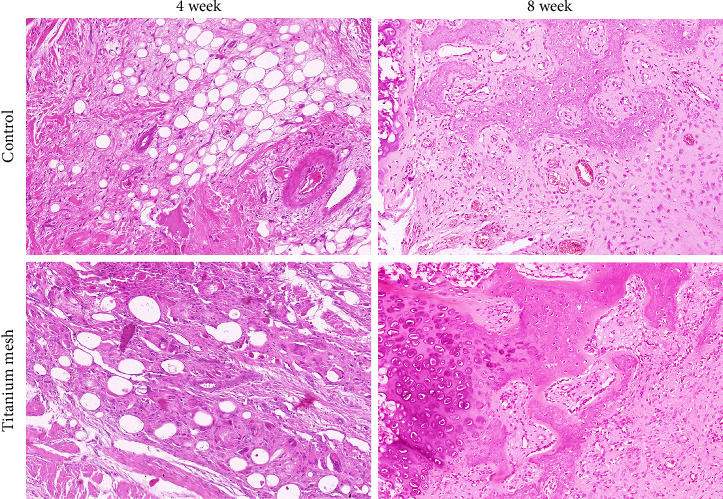
Bone histopathology (HE, 100×).

In order to observe the effect of titanium mesh on flail sternal healing, HE staining was performed on the rib tissue. The results showed that osteoblasts were abundant, chondrocytes were active, primary bone trabeculae formed, and a small amount of fibrous callus formed in both groups 4 weeks after operation (Figure 4). At 8 weeks after operation, the control group showed new bone tissue with irregular and sparse distribution of new bone trabeculae, while the experimental group showed bone trabeculae and bone callus formation with bone connection (Figure 4).

### 3.7. Expression of Bone Healing-Related Proteins in the Rib Tissues of Two Groups of Sheep

**Figure 5:**
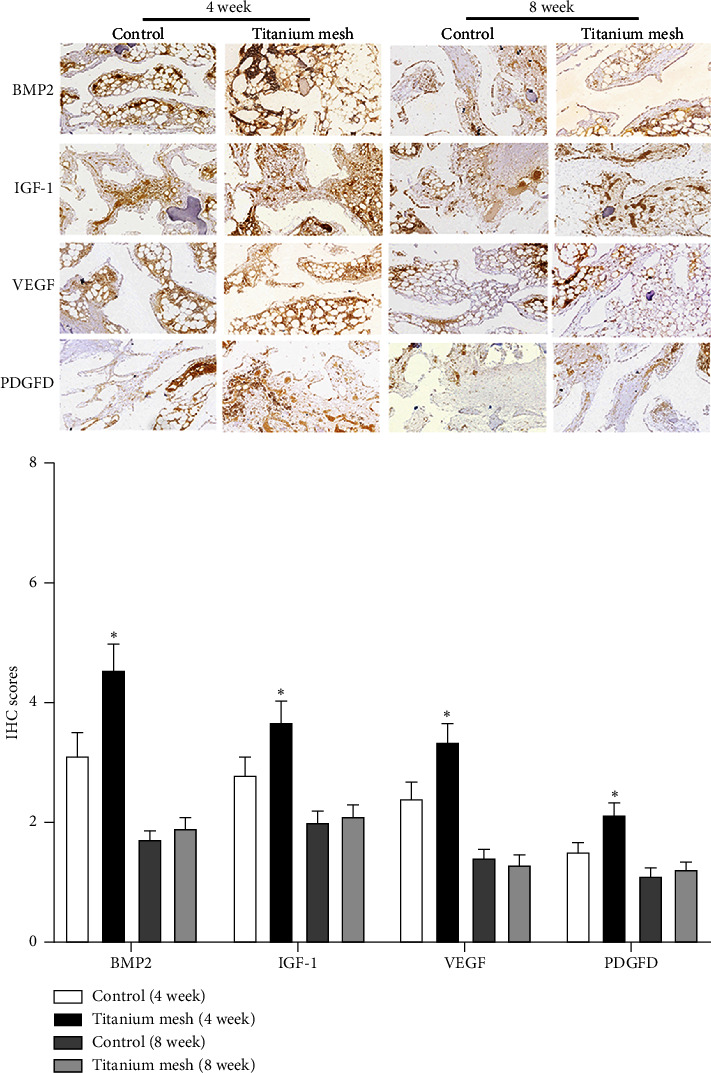
Expression of BMP2, VEGF, IGF-1, and PDGFD in sheep rib tissue detected by the IHC method (100×). Data represented as mean ± SD, *n* = 3. ⁣^∗^*p* < 0.05 compared with the control group.

Bone healing-related proteins play an important role in the process of fracture healing. Therefore, the expressions of BMP2, VEGF, IGF-1, and PDGFD in rib tissues were detected by the IHC method. The results showed that the IHC scores of BMP2, VEGF, IGF-1, and PDGFD in the rib tissue of the titanium mesh sheep were higher than those of the control group at 4 weeks after operation (Figure 5, *p* < 0.05). And the IHC scores of BMP2, IGF-1, VEGF, and PDGFD in the rib tissue of the two groups were not different at 8 weeks after operation (Figure 5, *p* > 0.05).

## 4. Discussion

Flail chest is one of the most common and severe closed chest traumas in clinical practice [9]. Due to multiple rib fractures, the injured chest wall loses bone support and becomes softened, resulting in the imbalance of thoracic pressure on both sides and the mediastinum swings from side to side with breathing, resulting in the obstruction of return blood flow, causing blood circulation disorders in patients, and leading to or aggravating shock and even death [10, 11]. At present, the treatment of flail chest stabilization chest mainly adopts chest strap compression bandaging to fix the chest wall softening area, chest wall protection plate fixation, traction with the external chest wall fixator with scarf clamp and mechanical ventilation, etc. [12]. When necessary, internal surgical fixation is given Refs. [13–15]. If the thorax cannot be stabilized as soon as possible and the softening range of the chest wall can be reduced, it is difficult to correct the mediastinal swing and the resulting respiratory and circulatory failure, so the injured need to rely on the ventilator for a long time, which greatly increases the cost and difficulty of medical treatment and the risk of death of the injured. However, the use of self-tissue materials for the reconstruction of bone thorax has the disadvantages of complicated operation, great trauma, and limited materials, which is not conducive to the early rapid reconstruction of bone thorax. The ideal artificial material has sufficient hardness, can effectively prevent the softening of the chest wall and abnormal respiratory movement, and has no adverse reactions and rejection when implanted in the body, and can be arbitrarily shaped according to the scope and shape of the chest wall softening during the operation, and the material is easy to be disinfected, sterilized, and preserved. Therefore, it is more conducive to the rapid treatment of flail chest in early treatment.

At present, it is found that the titanium material has important value in chest wall stability. The titanium plate is a flat-like structure made of pure titanium, which has high strength and rigidity and is of great value in chest wall stabilization [16, 17]. It has been applied in the treatment of flail chest [18–20]. However, the plasticity of the titanium plate is small and the cost is high. The titanium mesh is a mesh structure made of pure titanium, which is easier to make and adjust. The titanium mesh has good mechanical properties, and its high compressive strength can provide stable spatial support for the mesh. Due to its appropriate elasticity and plasticity, it can be shaped by bending to adapt to various bone defect forms and reduce the pressure on the mucosa [21–24]. Current cases and literature reviews have shown that the titanium mesh can be anchored at the base of bone defects to prevent fractures [25]. Previous studies have found that titanium mesh is a good choice for the treatment of comminuted mandibular fractures [26]. In this study, the application of titanium mesh in flail chest patients was analyzed, and the results showed that the mechanical ventilation time, drainage tube retention time, ICU observation time, and average hospital stay of titanium mesh patients were significantly shorter than those of control group, and the postoperative pain VAS score was significantly lower than that of the control group. PaO_2_ and CVP in the titanium mesh group were significantly higher than those in the control group after operation, while PaCO_2_, CI, and ELWI were significantly lower than those in the control group. The proportions of pulmonary ataxia, thoracic malformation, and delayed healing of the fractures in the titanium mesh group after surgery were significantly lower than those in the control group, and the indexes of pulmonary function FVC, FEV1, MVV, and SF-36 scores were significantly better than those in the control group, indicating that titanium mesh fixation effectively alleviated the pain in the recovery period of patients, accelerated the recovery of patients, and shortened the length of hospital stay of patients. It reduces the incidence of pulmonary ataxia, thoracic deformity, and delayed healing of fracture and has certain advantages in improving the hemodynamics and quality of life of flail chest patients and has positive significance for the recovery of lung function. Because the application of titanium mesh to fix flail chest rib fracture can restore the softened chest wall area and restore the normal chest shape, and it is not easy to loosen and can effectively stabilize the chest and achieve the purpose of treating flail chest.

To further verify the effect of titanium mesh on the flail chest, we used the titanium mesh on the flail chest sheep injury model. The results showed that the number of osteoblasts in titanium mesh sheep increased gradually, chondrocytes were active, and bony connections gradually appeared 4–8 weeks after operation. At the same time, IHC detection showed that the positive expressions of BMP2, IGF-1, VEGF, and PDGFD in the rib tissue of titanium mesh sheep were higher than those of the control group 4 weeks after operation. BMP2, as an important protein with osteogenic activity, can induce calcium to form calcium phosphate and deposit it in bone, which plays an important role in fracture healing [27]. IGF-1 is a multifunctional cell regulator with an active peptide in the chemical structure, which can not only promote the proliferation and differentiation of osteoblasts but also stimulate the proliferation of osteoblasts and the formation of the bone matrix [28, 29]. VEGF is an angiogenic factor that not only stimulates cardiovascular growth and increases vascular permeability but also plays a role in bone formation and repair by promoting local angiogenesis and osteoblast differentiation and participating in BMP-induced bone formation [30, 31]. PDGF is also an active polypeptide molecule and a stimulating source of mitosis for various cells. It has a chemotactic effect on mesenchymal cells, osteoblasts, fibroblasts, endothelial cells, and other cells and is one of the bone growth factors with the strongest chemotactic effect, playing an important role in fracture healing [32, 33]. These results indicate that titanium mesh can promote the bone healing of the flail chest and have a better fixing effect than the chest strap.

At the same time, there were some limitations in our study. First of all, we did not carry out sample size calculations. Second, the research results may be biased from the actual situation due to the insufficient sample size and the single-center study. Therefore, more samples needed to be included in the future to obtain more accurate conclusions. As a result, further studies need to be done to confirm these findings.

## 5. Conclusions

In summary, the treatment of flail chest with titanium mesh can improve pain, blood gas, hemodynamic indexes, and lung function and promote fracture healing and is a safe and effective treatment. At the same time, because of the simple operation technology, titanium mesh can be personalized shaped, which is conducive to rapid promotion and application.

## Figures and Tables

**Table 1:** Comparison of general data between the two groups.

Variable		Control group	Titanium mesh group	*p* values
Age (years)		45.75 ± 8.80	46.42 ± 9.00	0.856

Gender	Male	9 (75.00)	10 (83.33)	0.615
	Female	3 (25.00)	2 (16.67)	

BMI (kg/m^2^)		22.10 ± 1.78	22.75 ± 1.80	0.384

Cause of injury	Traffic accident	8 (66.67)	8 (66.67)	0.881
	Falling accidents	2 (16.67)	1 (8.33)	
	Blunt force trauma	1 (8.33)	2 (16.67)	
	Others	1 (8.33)	1 (8.33)	

Injured position	Unilateral	10 (83.33)	11 (91.67)	0.537
	Bilateral	2 (16.67)	1 (8.33)	

Associated injury	Craniocerebral injury	3 (25.00)	3 (25.00)	0.987
	Limb fracture	3 (25.00)	2 (16.67)	
	Abdominal organ injury	2 (16.67)	2 (16.67)	
	Pelvic fracture	1 (8.33)	1 (8.33)	
	No associated injury	3 (25.00)	4 (33.33)	

Number of injured ribs		3.3 ± 0.7	4.0 ± 1.0	0.058

*Note:* Data are presented as number, mean ± SD, and number (percentage) and analyzed using a two-sample independent *t* test or *χ*^2^ test.

**Table 2:** Comparison of clinical efficacy indexes between the two groups.

Variable		Control group	Titanium mesh group	*p* values
Mechanical ventilation time (d)		5.25 ± 1.22	3.83 ± 1.12	0.007

Drain indwelling time (d)		6.08 ± 1.00	3.50 ± 0.91	*p* ≤ 0.001

ICU observation time (d)		6.17 ± 1.27	4.58 ± 1.31	0.006

Hospital stays (d)		17.83 ± 2.41	12.75 ± 2.22	*p* ≤ 0.001

VAS score	Preoperative	7.58 ± 1.62	7.33 ± 1.61	0.709
	Postoperative 24 h	5.92 ± 1.17	4.50 ± 1.31	0.011
	Postoperative 48 h	4.58 ± 0.90	3.58 ± 1.1.08	0.022

*Note:* Data are presented as number and mean ± SD and analyzed using a two-sample independent *t* test.

**Table 3:** Comparison of blood gas indexes and hemodynamic indexes in different time periods between the two groups.

Variable		Control group	Titanium mesh group	*p* values
Preoperative	PaO_2_ (mm Hg)	64.47 ± 5.11	65.13 ± 5.54	0.765
	PaCO_2_ (mm Hg)	51.84 ± 4.59	51.19 ± 4.07	0.719
	SaO_2_ (%)	90.72 ± 3.43	91.42 ± 3.42	0.623
	CI (L·min^−1^·m^−2^)	3.36 ± 0.68	3.51 ± 0.60	0.566
	CVP (*p*/mm Hg)	6.08 ± 0.89	6.28 ± 0.91	0.577
	ELWI (mL·kg^−1^)	8.14 ± 1.75	8.33 ± 1.72	0.787

Postoperative 24 h	PaO2 (mm Hg)	75.24 ± 5.42	80.54 ± 5.91	0.032
	PaCO_2_ (mm Hg)	47.42 ± 3.33	44.46 ± 3.48	0.045
	SaO_2_ (%)	92.24 ± 3.71	92.14 ± 3.56	0.948
	CI (L·min^−1^·m^−2^)	4.66 ± 0.73	4.08 ± 0.59	0.040
	CVP (*p*/mm Hg)	7.84 ± 1.52	10.06 ± 2.26	0.010
	ELWI (mL·kg^−1^)	13.17 ± 1.54	11.28 ± 1.80	0.012

Postoperative 48 h	PaO_2_ (mm Hg)	81.69 ± 6.03	88.21 ± 7.66	0.030
	PaCO_2_ (mm Hg)	40.49 ± 2.75	37.11 ± 3.94	0.023
	SaO_2_ (%)	93.60 ± 3.9	94.64 ± 2.96	0.466
	CI (L·min^−1^·m^−2^)	4.86 ± 0.78	4.21 ± 0.59	0.033
	CVP (*p*/mm Hg)	9.63 ± 1.96	11.88 ± 1.86	0.009
	ELWI (mL·kg^−1^)	13.83 ± 2.52	11.69 ± 2.46	0.047

*Note:* Data are presented as number and mean ± SD and analyzed using a two-sample independent *t* test.

**Table 4:** Comparison of postoperative CT results and lung function between the two groups.

Variable		Control group	Titanium mesh group	*p* values
Postoperative 1 month	Atelectasis	8 (66.67)	3 (25.00)	0.041
	Thoracocyllosis	9 (75.00)	4 (33.33)	0.041
	Consolidation tardive	10 (83.33)	5 (41.67)	0.035
	FVC (L)	2.03 ± 0.29	2.34 ± 0.28	0.013
	FEV1 (L)	1.55 ± 0.23	1.83 ± 0.29	0.017
	MVV (L/min)	67.63 ± 5.29	73.45 ± 4.08	0.006

Postoperative 2 months	Not full expansion	6 (50.00)	1 (8.33)	0.025
	Thoracocyllosis	7 (58.33)	2 (16.67)	0.035
	Consolidation tardive	8 (66.67)	3 (25.00)	0.041
	FVC (L)	2.48 ± 0.47	2.87 ± 0.46	0.049
	FEV1 (L)	2.11 ± 0.31	2.43 ± 0.40	0.037
	MVV (L/min)	76.34 ± 6.54	83.03 ± 7.78	0.033

*Note:* Data are presented as number and mean ± SD and number (percentage) and analyzed using a two-sample independent *t* test or *χ*^*2*^ test.

**Table 5:** Comparison of postoperative SF-36 scores between the two groups.

Variable	Control group	Titanium mesh group	*p* values
PF	55.58 ± 6.30	61.67 ± 7.02	0.036
RP	56.08 ± 6.39	63.42 ± 6.68	0.012
BP	49.92 ± 5.55	59.58 ± 6.10	0.001
GH	52.67 ± 5.57	61.08 ± 7.49	0.005
VT	56.92 ± 5.57	62.75 ± 5.68	0.019
SF	64.92 ± 6.60	71.58 ± 7.89	0.035
RE	66.25 ± 6.49	70.67 ± 7.12	0.065
MH	68.42 ± 6.43	74.67 ± 7.22	0.036

*Note:* Data are presented as number and mean ± SD and analyzed using a two-sample independent *t* test.

Abbreviations: BP, bodily pain; GH, general health; MH, mental health; PF, physiological function; RE, role emotional; RP, role physical; SF, social function; VT, vitality.

**Table 6:** Comparison of blood gas indexes at different times between the two groups of sheep.

Variable		Control group	Titanium mesh group	*p* values
Preoperative	PaO_2_ (mm Hg)	87.45 ± 7.52	88.12 ± 7.53	0.918
	PaCO_2_ (mm Hg)	40.82 ± 3.06	41.57 ± 3.11	0.781
	SaO_2_ (%)	98.64 ± 4.94	99.12 ± 5.17	0.913

Postoperative 24 h	PaO_2_ (mm Hg)	63.15 ± 5.16	68.54 ± 5.72	0.292
	PaCO_2_ (mm Hg)	52.39 ± 3.51	49.47 ± 3.26	0.381
	SaO_2_ (%)	92.53 ± 4.37	94.12 ± 4.28	0.676

Postoperative 48 h	PaO_2_ (mm Hg)	70.17 ± 5.64	79.92 ± 5.97	0.109
	PaCO_2_ (mm Hg)	46.87 ± 3.14	42.16 ± 2.98	0.133
	SaO_2_ (%)	97.61 ± 5.64	98.89 ± 5.73	0.470

*Note:* Data are presented as number and mean ± SD and analyzed using a two-sample independent *t* test.

## Data Availability

The data that support the findings of this study are available from the corresponding author upon reasonable request.
